# Strategies to functionalize extracellular vesicles against HER2 for anticancer activity

**DOI:** 10.20517/evcna.2022.07

**Published:** 2022-04-18

**Authors:** Elena Gurrieri, Vito Giuseppe D’Agostino

**Affiliations:** Department of Cellular, Computational and Integrative Biology, University of Trento, Trento 38123, Italy.

**Keywords:** Extracellular vesicles, human epidermal growth factor receptor 2 (HER2/ERBB2), EV engineering, fusion proteins

## Abstract

Cell-secreted extracellular vesicles (EVs) are membranous particles highly heterogeneous in size and molecular cargo. Comprehensively, released EV sub-populations can show a wide range and selection of different protein, RNA, and lipid species, complementing cell communication signals. Recently, EVs represent a new source for developing targeted delivery systems. EVs are stable in biofluids, intrinsically biocompatible with low immunogenicity, and capable of transferring cargo molecules into “recipient” cells. The immune-mediated recognition represents a popular approach to functionalize and direct EVs towards receptor-positive cell populations. The human epidermal growth factor receptor 2 (HER2, also known as *neu* or ERBB2) is a tyrosine kinase of clinical relevance, targeted by several available antibodies, and a model receptor used to test the biodistribution and anticancer activity of bioformulations, including EVs. Here, we focus on recent strategies adopted for EV functionalization with fusion ligands able to recognize HER2, covering the enhanced expression of membrane-fusion proteins in “EV-donor” cells as well as post-isolation EV-surface modifications.

## INTRODUCTION

The establishment of programmable and versatile delivery systems that could control the dosage of the therapeutic agent in the tumor site and bypass biological barriers represents a current challenge in biomedical research. Extracellular vesicles (EVs) can serve as a new biological source for targeted drug delivery and the development of nanoparticle-based technologies^[1,2]^. EVs are cell-secreted membranous particles highly heterogeneous in size and molecular cargo. Ranging from nanometer to micrometer scale, EVs are classified as exosomes or microvesicles according to the cellular pathways responsible for their release from the endosomal system or the plasma membrane, respectively^[3]^. As cell-derived material, secreted EV sub-populations can show a wide range and selection of different species of proteins, RNA, and lipids, complementing cell communication signals^[4,5,6]^. Substantiated by the observed stability in biofluids, intrinsic biocompatibility, low immunogenicity, and capacity to convey the cargo molecules into “recipient” cells, EVs have been proposed as advantageous delivery vehicles compared to liposomes or other synthetic biomaterials^[7,8]^. For example, EVs were successfully employed *in vitro* and *in vivo* for miRNA delivery, siRNA-based gene silencing, or shuttling mRNAs encoding for reporter proteins^[9,10,11]^. 

The immune-mediated interaction is commonly exploited to direct EVs towards specific membrane receptors on the desired cell populations^[12]^. The family of epidermal growth factor receptors (EGFRs) includes well-known functional ligands and clinically relevant biomarkers in solid tumors, such as breast, ovarian, and stomach cancers. The proto-oncogene human epidermal growth factor receptor 2 (HER2, also known as *neu* or ERBB2) is a member of tyrosine kinase receptors involved in cell differentiation, proliferation, and migration^[13]^. Overexpression of this glycoprotein can be due to *HER2* gene amplification or aberrant protein expression in tumor tissues^[14,15,16]^. Since the secreted EV sub-populations display proteins that mirror the plasma-membrane identity of the donor cells, the HER2 receptor has been detected in EVs recovered by different methods, from media of cultured cells or the blood of patients with HER2-positive tumors. Indeed, Nanou *et al.*^[17]^ (2020) encouraged the screening of vesicular HER2 in clinical settings as they found blood circulating EVs extremely informative on the presence of HER2-positive primary tumors and proposed them as valuable prognostic factors complementing circulating tumor cells (CTCs)^[17]^. Kim *et al.*^[18] ^(2020) described a positive correlation between cellular HER2 and receptor enrichment in the smallest EV fractions, confirming the presence of HER2 in EVs circulating in breast cancer patients with different tumor stages^[18]^. Vesicular HER2 was also detected in human serum using a primary antibody with the same variable region of trastuzumab^[19,20]^, an antibody under clinical use since 1998 for treating HER2-positive breast cancer patients^[13]^. Interestingly, Quinn *et al.*^[21]^ (2021) reported that EVs released from cells overexpressing HER2 could horizontally transfer the protein to receptor-negative cells, sensitizing them to paclitaxel^[21]^.

Different monoclonal antibodies or fusion moieties have been developed to target the extracellular domain of HER2^[22]^, leading to cytostatic effects or cell death when in conjugation with cytotoxic compounds^[23,24,25]^. These moieties were explored for their targeted delivery potential within different bioformulations, including unilamellar liposomes loaded with chemotherapy agents^[26]^, gold nanoparticles^[27]^, or silk nanospheres^[28]^.

In this review, we summarize the different strategies of EV-functionalization with ligands able to recognize the HER2 receptor, including seminal examples designed on EGFR, ranging from the enhanced expression of membrane-fusion proteins in EV-donor cells to post-isolation approaches.

## STRATEGIES TO FUNCTIONALIZE EVs BY MANIPULATING DONOR CELLS

The manipulation of specific cells conceived as EV-producers is one of the most common strategies adopted to enrich vesicle sub-populations with specific surface proteins. This approach finds a rationale, for example, with chimeric antigen receptor (CAR)-T cells which can be directed against a tumor-expressing antigen. Indeed, there is evidence that heterogeneous EVs deriving from CAR-T cells, over-expressing an anti-HER2 ligand, can penetrate and induce apoptosis in target cells, albeit with a delayed timing compared to donor cells^[29]^.

As already demonstrated for the anti-EGFR strategy, donor cells can be used to transiently or stably overexpress multi-domain proteins subsequently detected on secreted EVs. These constructs generally encode for a receptor transmembrane (TM) domain, a linker, and an antibody-derived targeting moiety. This last portion can also be fused with a reporter, such as the green fluorescent protein (GFP)^[30]^. Leading examples of HER2-targeting strategy include the lysosome-associated membrane protein 2b (LAMP2b), the platelet-derived growth factor receptor domain (PDGFR TM), and the membrane-associated C1-C2 domains of lactadherin.

### LAMP2b

Limoni *et al.*^[31] ^(2019) isolated exosomes from HEK293T cells expressing a fusion protein constituted by LAMP2b followed by designed ankyrin repeat protein (DARPin) G3, a 14 kDa-engineered peptide targeting HER2. The authors observed that the concentration of particles used to treat recipient cells was crucial for targeting HER2-positive SKBR3 cells compared to HER2-negative MDA-MB-231 cells. In addition, the isolated exosomes were able to deliver siRNAs specifically, ultimately impacting the levels of *TPD52* in recipient cells^[31]^. The same group used the construct to transduce bone marrow mesenchymal stem cells (MSCs) and isolate exosomes that were electroporated in the presence of doxorubicin (exo-DOX). In this case, LAMP2b-DARPin-exo-DOX confirmed a specific targeting profile up to 0.1 μg/μL, while not at 0.2 μg/μL, and induced death of TUBO cells to a greater degree than free doxorubicin^[32]^.

In the study by Molavipordanjani *et al.*^[33] ^(2020), radiolabeled 99mTc-DARPins were used to decorate exosomes with an affinity for HER2. They tested the uptake ratio of their preparations using SKOV-3, MCF-7, U87-MG, HT-29, and A549 cell lines characterized by different levels of HER2 expression. They profiled an accumulation of particles proportional to the higher expression of the receptor (SKOV-3 cells). The authors also evaluated the biodistribution of their particles injected in normal and SKOV-3 xenografted nude mice and reported a high liver uptake at one hour, which gradually decreased at four hours. In addition, the intestines and kidneys showed a consistent level of radioactivity, while it decreased in a time-dependent manner in other tissues, including the spleen, lungs, and blood. The same pattern of biological distribution, including accumulation in the tumor site, was observed in the xenografted mice subjected to tumor tissue visualization by planar imaging^[33]^.

A LAMP2b-directed fusion protein approach was also adopted by Liang *et al.*^[34] ^(2020), who fused a HER2-binding affibody (or antibody mimetic) at the C-terminus with the addition of GFP. They reported the complete sequence of the fusion protein, including a flexible peptide linker (GGGGS)_3_, and exploited exosomes to co-deliver oligonucleotides and drugs to HER2-positive colorectal cancer cells. Besides the cell-targeting properties of the preparations reaching HER2-mcherry-SGC-7901 cells or the tumor *in vivo*, encapsulated 5-fluorouracil (5-FU) together with miR-21 inhibitor nucleotide (miR-21i) were found to be more effective than exosomes loaded with either miR-21i or 5-FU alone^[34]^.

### PDGFR TM

Another interesting strategy exploited EVs co-expressing CD3 and HER2 ligands to enhance the proximity of T cells to tumor cells and elicit an anticancer effect in the presence of non-activated human peripheral blood mononuclear cells (PBMCs) *in vivo*^[35]^. In this study, the authors exploited the human PDGFR TM domain fused to an antibody single-chain variable fragments (scFv) connected by a (GGGGS)_3_ linker in the CD3/HER2 bi-specific chimeric protein. By confirming cellular uptake *in vitro*, the authors demonstrated cytotoxic activity selectively enhanced in HER2-expressing breast cancer cells mediated by T cell activation compared with native vesicles still isolated by ultracentrifugation. The same qualitative data were obtained using mouse xenografts with no significant effects reported in total body weight or specific organ damage, especially liver or kidney^[35]^.

### Lactadherin C1-C2 domains

Longatti *et al.*^[36]^ (2018) transduced HEK293 cells to express anti-HER2 scFvs fused to C1-C2 domains of lactadherin, which associate with phosphatidylserine (PS) in exosome fractions. The authors used three different scFvs covering a range of high (Kd~15 pM), intermediate (Kd~1 nM), and low (Kd~317 nM) affinity for HER2. They compared the different preparations by also varying the HER2 expression level on recipient cells, finding that both high-affinity scFv and high receptor expression were parameters positively influencing the selective uptake^[36]^. Another targeting moiety to direct exosomes against the receptor consisted of two copies of HER2 ligand still fused to the C-terminal C1-C2 domains^[37]^. The authors reported ELISA experiments showing a four-fold enhanced binding of recovered exosomes compared to untargeted ones. *In vivo* experiments demonstrated a tumor volume reduction of implanted SKOV-3 cells upon injection of targeting exosomes carrying a HER2-downregulating miRNA^[37]^. The lactadherin C1-C2 domains fused to anti-HER2 scFv were used by Forterre *et al.*^[38]^ (2020) to deliver prodrugs that exert cytotoxic activity in recipient cells. The authors showed a near-complete growth arrest of human HER2-positive breast cancer xenografts upon systemic administration in athymic mice, with no reported injury to other tissues and absence of “off-target mRNA delivery”^[38]^.

## STRATEGIES TO FUNCTIONALIZE EVs POST-ISOLATION

Since biochemical strategies focused on HER2 emulate approaches already presented for targeting EGFR, we include the most recent post-isolation designs to direct vesicles against EGFR. Seminal approaches include the use of lactadherin C1-C2 domains, protein ligation, and antibody-receptor binding.

In the study by Kooijmans *et al.*^[39] ^(2016), the nanobody EGa1 was conjugated to PEG-phospholipid micelles, subsequently incorporated into EVs, and then purified by size-exclusion chromatography. The authors reported that PEGylation increased circulation time in the blood of tumor-bearing mice and presented this method as a versatile tool to increase the stability of targeting EVs^[39]^. A different study followed in 2018 and reported EVs decorated with C1-C2 fusion proteins^[40]^. The authors expressed in HEK293 a fusion protein connecting the EGa1 sequence to the PS-binding domains of lactadherin (C1-C2) via a GGGS2 linker. The EGa1-C1-C2 protein was generated together with R2-C1-C2 protein as a negative control, and the native recombinant proteins were purified from cell culture media, still retaining binding activity. The final preparation was obtained by incubating fusion proteins with PS-bearing EVs following the nanobody:EVs ratio of ng:µg. Exosomes isolated from red blood cells (RBCs) and mouse neuroblastoma cells were reported to maintain their size and integrity after decoration with the fusion proteins. The authors showed enhanced specific binding (incubation at 4 °C for 1 h) and uptake (incubation at 37 °C for 4 h) by receptor-positive cells^[40]^.

Wang *et al.*^[41]^ (2018) provided an example of enzyme/prodrug therapy mediated by targeted EVs. They designed a chimeric protein containing a high-affinity anti-HER2 scFv antibody fused to lactadherin C1-C2 domains. To engineer EVs, they showed that post-isolation incubation of EVs from HEK293 with the protein alone was more efficient than HEK293 transfection with the plasmid encoding for the protein. They showed that the PKH26-labeled targeted EVs could bind to HER2-positive cells with a selective cytotoxic profile *in vitro* when combined with the mRNA of the enzyme responsible for the prodrug activation. In addition, their preparations inhibited HER2-positive tumor xenografts growth in mice^[41]^. 

Other authors exploited the protein ligase OaAEP1 to conjugate a biotinylated EGFR-targeting peptide (Biotin-YHWYGYTPQNVI-GGGGS-NGL)^[42]^ to EVs recovered from human red blood cells (RBCEVs)^[43]^. They proved an increased internalization of functionalized EVs in EGFR-positive cells after 2 h of incubation at 37 °C. The authors performed a similar enzymatic ligation to conjugate RBCEVs with an α-EGFR camelid biparatopic nanobody (VHH) and reported the importance of a linker peptide with the ligase binding site at the C-terminus (GGG-Myc-GLPETGG), necessary as a bridge to reduce the steric hindrance between the VHH and the RBCEV surface. *In vivo* experiments with immunodeficient NOD scid gamma (NSG) mice bearing luciferase-mCherry-H358 lung tumors demonstrated that tail vein-injected EVs preferentially accumulated in the lung, as expected, as compared to the liver, and enhanced the effect of paclitaxel, pre-loaded through sonication^[43]^.

Focusing directly on the HER2 receptor, Sato *et al.*^[44]^ (2016) produced hybrid particles through membrane fusion between exosomes derived from a mouse macrophage cell line and liposomes after up to 10 freeze-thaw cycles. They used zwitterionic, cationic, or anionic lipids to explore different lipidic compositions and found that exosomes from mouse fibroblast sarcoma cells, overexpressing HER2, maintained the receptor expression after fusion with PEGylated anionic lipids. These preparations showed increased uptake in HeLa cells^[44]^.

A different approach was proposed by Barok *et al.*^[45] ^(2018), who decorated exosomes from HER2-positive cancer cells with the antibody–drug conjugate trastuzumab emtansine (T-DM1). They isolated EVs from the secretome of several HER2-positive cancer cell lines and incubated them with T-DM1 (25 ug/mL at 4 °C for 30 min). Confocal microscopy imaging showed that EV preparations, exposed overnight, preferentially entered HER2-positive cells, inhibiting proliferation and inducing caspase activation^[45]^.

## CONCLUSION

EVs represent a valuable source in liquid biopsy studies as cargo of clinically relevant biomarkers, including HER2 and EGFR. Recent reports also indicate that EVs decorated with anti-HER2 ligands hold promise for targeted therapy, expanding the repertoire of nanoparticle-based technologies^[46]^.

**Figure 1:**
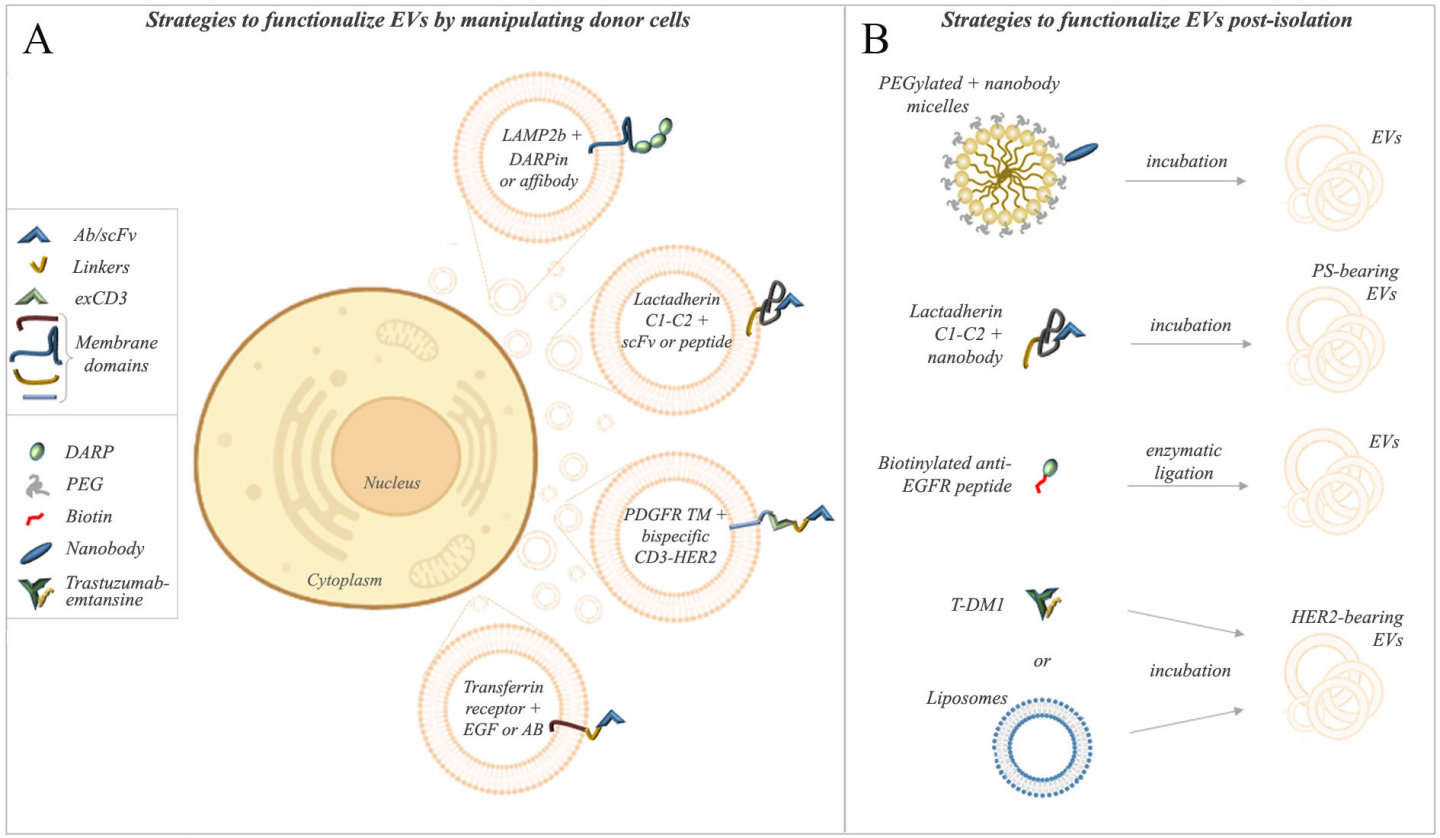
Strategies for EV targeting against HER2/EGFR. Pre-isolation strategies (A): manipulation of EV-producing cells to enrich targeting proteins in EVs. TM proteins (LAMP2b), TM domains (PDGR, transferrin receptor), or membrane-associated domains (C1-C2 of lactadherin) are exploited to anchor the targeting moieties to the EV membrane. Post-isolation strategies (B): EVs are directly functionalized by incubation with nanobodies or targeting peptides alone or fused to membrane-associated domains (C1-C2 of lactadherin). Post-isolation modifications also include the fusion of EVs with micelles or liposomes, forming hybrid particles. Both pre- and post-isolation approaches include scFvs, affibodies, nanobodies, DARPins, or peptides as targeting moieties. The figure was partially created on www.biorender.com.

In Figure 1, we summarize the pre- and post-isolation strategies thus far presented to direct EVs against HER2/EGFR receptors. The lactadherin C1-C2 domains emerged as the most versatile system used either in pre- or post-isolation strategies. However, we could not determine comparative relationships based on the yield of specific EV sub-populations, relative *in vitro* and *in vivo* stability, HER2-binding efficiency, or tissue-specific EV internalization rates. Table 1 highlights the liquid biopsy studies reporting the detection of blood circulating HER2-positive EVs in breast cancer patients and the outcomes described in preclinical models with the corresponding targeting strategy. In xenograft studies, the LAMP2b- and lactadherin C1-C2-based strategies effectively inhibited tumor growth with a certain degree of tissue specificity.

**Table 1:** Summary of the liquid biopsy studies reporting the detection of blood circulating HER2-positive extracellular vesicles (EVs) in breast cancer patients and the outcomes described in preclinical models with the corresponding targeting strategy

**Source**	**Liquid biopsy studies**	**Targeting strategy**	**Preclinical model**	**Outcome description**	**Ref.**
Metastatic breast cancer patients with HER2-positive tumors	Blood			Detection of HER2-positive EVs and prognostic significance	Nanou *et al.*^[17]^, 2020
Breast cancer patients at different tumor stage	Blood			Detection of HER2-positive EVs	Kim *et al.*^[18]^, 2020
Breast cancer patients	Blood			Detection of HER2-positive EVs	Jiang *et al.*^[19]^, 2019; Ciravolo *et al.*^[20]^, 2012
		PDGFR TM + GE11	Breast cancer xenograft in RAG2^–/–^ mice	Migration of exosomes to tumor tissues and inhibition of tumor development	Ohno *et al.*^[12]^, 2013
		Targeting peptide ligated to EV-membrane proteins	Mice bearing luciferase-mCherry-H358 EGFR-positive lung tumors	Tail vein-injected EVs preferentially accumulated in the lung more than in the liver; enhanced the efficacy of paclitaxel	Pham *et al.*^[43]^, 2021
		GPI conjugated to EGa1 nanobody	A431-xenografted mice	Increased EV circulation time	Kooijmans *et al.*^[39]^, 2016
		LAMP2b + DARPin	TUBO-xenografted mice	Selective tissue distribution and reduction of the tumor growth rate	Gomari *et al.*^[32]^, 2019
		LAMP2b + DARPin G3	SKOV-3-xenografted mice	Tumor site accumulation together with other organs	Molavipordanjani *et al.*^[33]^, 2020
		LAMP2 + affibody	Colon cancer xenografts in BALB/c mice	Significant reduction of tumor growth	Liang *et al.*^[34]^, 2020
		PDGFR TM + CD3/HER2 ligands	Mice bearing HCC 1954 tumors, engrafted with human PBMCs	Pharmacokinetic profile; significant inhibition of tumor growth; no systemic cytotoxicity; significant T cell infiltrations in the tumor site	Shi *et al.*^[35]^, 2020
		Lactadherin C1-C2 + targeting peptide	Mice with HER2-negative or -positive tumor xenografts	Reduction of HER2-positive tumor volume	Wang *et al.*^[37]^, 2020
		Lactadherin C1-C2 + scFv	BALB/C athymic mice with human HER2-positive BT474 xenografts	Growth arrest of xenografts; no reported injury to other tissues	Forterre *et al.*^[38]^, 2020
		Lactadherin C1-C2 + scFv ML39	Mice with HER2-positive BT474 xenografts	Inhibition of HER2-positive tumor growth; EV-mediated delivery of exogenous mRNA	Wang *et al.*^[41]^, 2018

Considering the qualitative proof-of-principles reported with both *in vitro* and *in vivo *models using cell-secreted EVs, the preparation of the vesicle suspension represents a relevant aspect. A repeatable and homogenous suspension of the bioformulation is a crucial requirement to develop EV-based nanotechnologies. On the one side, we cannot exclude that the high heterogeneity of cell-secreted EVs could be directly responsible for their stability in circulation or the biological effects exerted in recipient cells due to a sort of induced “parallel, cumulative signaling/internalization”. On the other side, technical challenges exist in establishing single-step, multi-parametric methodologies that efficiently separate vesicle sub-populations characterized by homogeneous biophysical (size/concentration distribution) and biological (lipid/protein/nucleic acid content) profiles.

After manipulating EV-donor cells, one of the relevant challenges is the efficient recovery of the desired EV sub-populations. Conversely, the post-isolation methods often suffer from an induced increment of size and/or particle aggregation. A deeper characterization and separation of EV sub-populations using isogenic cell models could improve our knowledge of the internalization performance or the degradation/recycling pathways upon endocytosis. A direct, systematic comparison among the designed tools will contribute knowledge on improving the preparations’ stability, how the receptor’s expression level influences the EV up/intake, or the advantages conferred by specific linkers. In this regard, the presence of linkers of different lengths that confer distance and flexibility to the targeting moiety on the EV surface has been evaluated in synthetic immunoliposomes^[26]^ as well as engineered EVs, favoring the particle uptake^[43,34,40]^. Nevertheless, the storage, freezing cycles, and potential cryopreserving agents could be relevant to correlating morphological changes and quality of proteins/RNAs with bioactivity^[47,48]^.

We believe these are fundamental aspects contributing to the development of promising routes for establishing EV-based, innovative delivery systems.

## DECLARATIONS

### Copyright

© The Author(s) 2022.
